# Giant sporadic renal angiomyolipoma as a cause of a massive abdominopelvic mass: A case report

**DOI:** 10.1097/MD.0000000000046912

**Published:** 2026-01-16

**Authors:** Lingtao Yan, Xingzhi Li, Ting Yuan, Yu Wang

**Affiliations:** a Department of Urology, 363 Hospital, Chengdu, China; b Department of Pathology, Chengdu Sixth People’s Hospital, Chengdu, China.

**Keywords:** arterial embolization, giant renal angiomyolipoma, massive abdominopelvic mass, radical nephrectomy

## Abstract

**Rationale:**

Renal angiomyolipoma (RAML) is a benign solid tumor that is typically small and asymptomatic. We present a rare case of a giant sporadic RAML to contribute to the understanding of the diagnosis and management of massive abdominopelvic masses.

**Patient concerns:**

A 35-year-old woman was admitted to our hospital after a magnetic resonance imaging scan at a local county hospital revealed a massive abdominopelvic mass. She had previously undergone vaginal delivery and had no significant medical history.

**Diagnoses:**

Physical examination revealed a large, firm, fixed mass in the left abdomen and pelvis. An abdominal computed tomography scan confirmed the presence of a massive tumor originating from the left kidney. Postoperative histopathological examination confirmed the diagnosis of RAML.

**Interventions:**

The tumor was successfully resected via open radical nephrectomy with minimal blood loss following preoperative arterial embolization.

**Outcomes:**

The patient recovered well and reported no postoperative complications during follow-up.

**Lessons:**

Sporadic RAMLs exceeding 30 cm in size are exceptionally rare. This case demonstrates the successful surgical management of a giant RAML causing a massive abdominopelvic mass, with controlled bleeding due to preoperative embolization. Additional case reports are necessary to refine treatment approaches for giant RAML.

## 1. Introduction

Angiomyolipoma (AML) is a benign solid tumor composed of varying proportions of fat, smooth muscle and blood vessels, most commonly found in the kidneys. It is classified into sporadic renal angiomyolipoma (RAML) and tuberous sclerosis complex (TSC)-associated RAML.^[1]^ Sporadic RAML is typically small and asymptomatic, often detected incidentally during routine health examinations.^[2]^ While multiple treatment options exist, surgery remains the primary approach for giant RAML.^[3]^ However, the large size of these tumors poses significant therapeutic challenges. Here, we report an exceptionally rare case of a woman with a giant sporadic RAML presenting as a massive abdominopelvic mass and describe a novel treatment approach for such cases.

## 2. Case description

A 35-year-old woman was referred to our hospital (363 Hospital) following the incidental discovery of a massive abdominopelvic mass originating from the left kidney on magnetic resonance imaging (MRI) at a local county hospital. Her only subjective complaint was recent weight gain; she denied hematuria, pain, or other symptoms. Her medical history was unremarkable except for a prior vaginal delivery. Physical examination revealed a large, firm, fixed mass in the left abdomen without rebound tenderness or muscle guarding.

**Figure 1.**
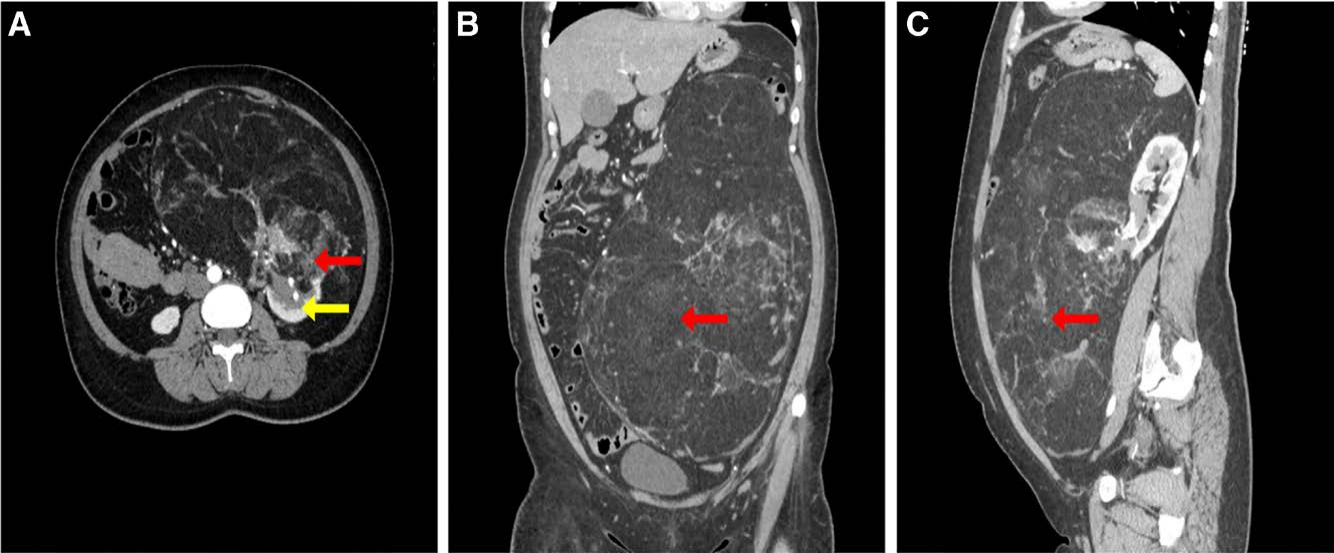
Preoperatively CT scan. (A) Axial, (B) coronal, (C) sagittal. The illustration shows the tumor (red arrow) and the left kidney (yellow arrow). CT = computed tomography.

Abdominal contrast-enhanced computed tomography (CT) demonstrated a 32.2 cm × 23.0 cm × 14.5 cm fat-density mass in the left abdominopelvic region, contiguous with the low pole of the left kidney (Fig. 1). Brain MRI showed no features of TSC. Laboratory investigations, including renal function tests with normal bilateral glomerular filtration rates, were unremarkable. Given the tumor’s enormous size precluding partial resection, we obtained informed consent for open radical nephrectomy (RN) following preoperative embolization.

**Figure 2.**
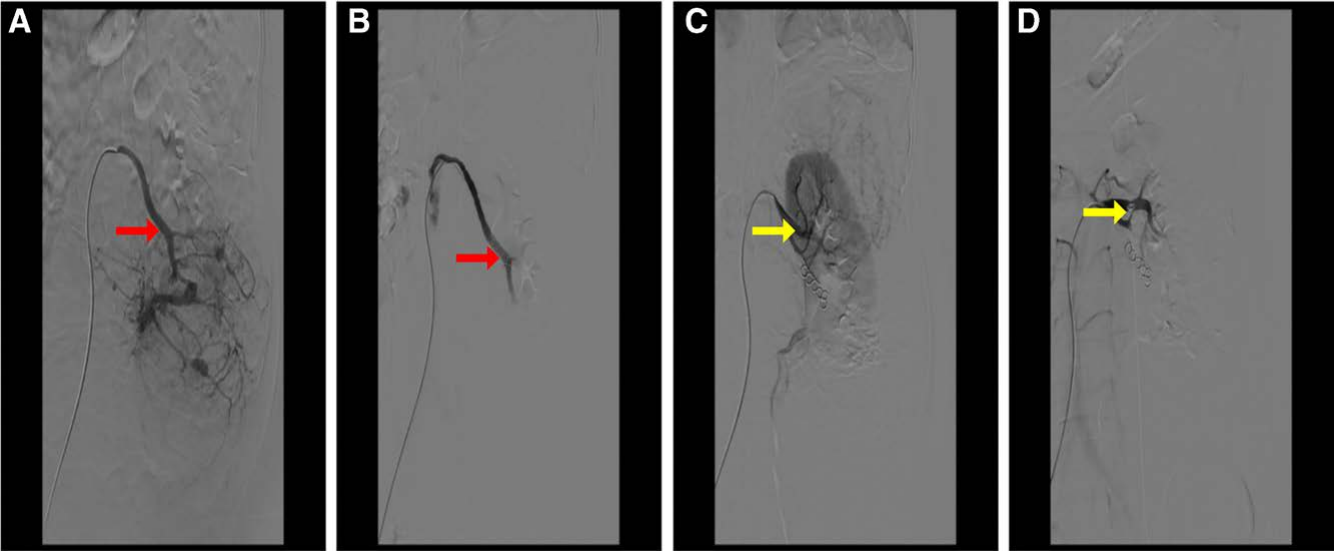
Angiography and embolization. (A) Prior to renal artery embolization, (B) following renal artery embolization, (C) prior to accessory renal artery embolization, (D) following accessory renal artery embolization. The illustration depicts the renal artery (red arrow) and the accessory renal artery (yellow arrow).

**Figure 3.**
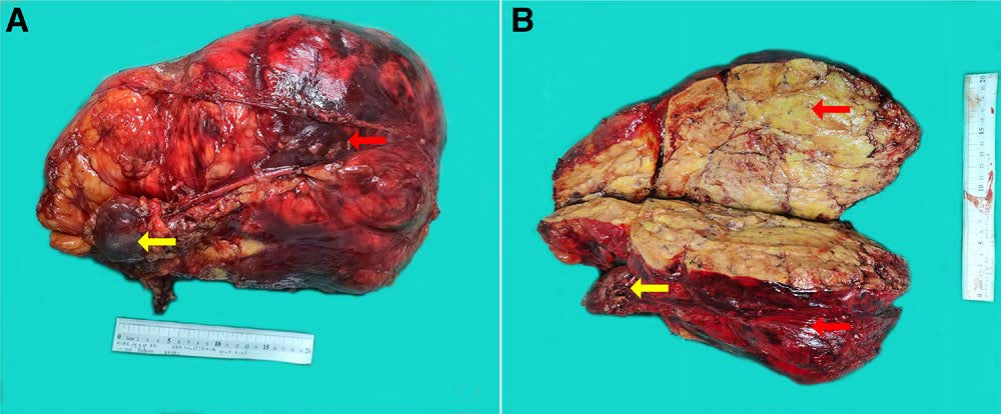
(A, B) Macroscopic examination of the specimen reveals a large tumor (red arrow) and a reduced-sized left kidney (yellow arrow).

Preoperatively, angiographic embolization was performed to occlude the left renal artery and tumor-feeding vessels, including the left accessory renal artery, selective branches of the left renal artery and a contributing branch from the inferior mesenteric artery (Fig. 2). Immediately following embolization, the patient underwent open RN via an *L*-shaped incision extending from the xiphoid process to the umbilicus and continuing to the left lower abdominal quadrant. The resected specimen measured 36 cm × 26 cm × 13 cm with a characteristics yellow cut surface and weighed 6.5 kg (Fig. 3).

**Figure 4.**
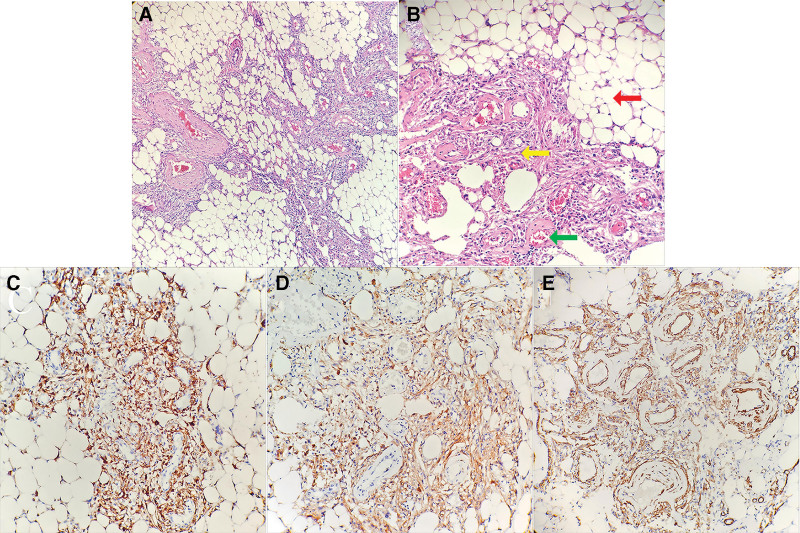
Histopathological examination of the lesion reveals the presence of 3 distinct components: adipose tissue (red arrow), smooth muscle (yellow arrow), and blood vessels (green arrow). (A) HE staining ×100, (B) HE staining ×200. Immunohistochemical staining of the lesion demonstrates positive expression of specific markers. (C) HMB-45, (D) Melan-A, (E) SMA. HE = hematoxylin-eosin, HMB-45 = human melanoma black 45, SMA = smooth muscle actin immunohistochemistry.

**Figure 5.**
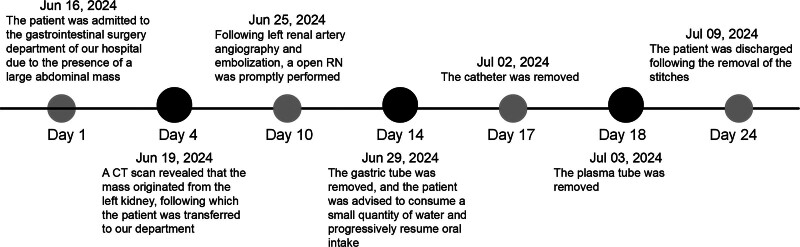
A timeline diagram.

Histopathological examination confirmed the diagnosis of AML, with immunohistochemical staining demonstrating the following profile: positive markers: human melanoma black 45, Melan-A, smooth muscle actin immunohistochemistry; low proliferative index: Ki-67 (1–2%); and negative markers: P-CK, PAX-8, S-100, Desmin (Fig. 4). These findings definitively established the diagnosis of RAML. The patient’s postoperative course was unremarkable, with suture removal performed prior to discharge (Fig. 5). At 1-year follow-up, the patient remained asymptomatic with no evidence of complications. She expressed satisfaction with her weight loss and reported no additional concerns.

## 3. Discussion

Sporadic RAML typically presents as a small benign tumor, with an average size of 1.08 ± 0.58 cm and an annual growth rate of approximately 0.19 cm (5%).^[2,4]^ Tumor size correlates strongly with clinical manifestations, as lesions exceeding 4 cm in diameter carry a significant risk of spontaneous rupture and life-threatening hemorrhage.^[3]^ Imaging characteristics vary by modality: ultrasound: classic RAMLs with fat components appear hyperechoic, though this feature cannot reliably differentiate them from hyperechoic renal cell carcinomas; CT: the gold standard for diagnosing classic fat-containing RAMLs, but limited in identifying fat-poor variants which may mimic renal cell carcinoma; and MRI: superior for evaluating fat-poor RAMLs through characteristic “India ink artifact” at fat – water interfaces, though its clinical utility is constrained by higher cost and limited availability compared to CT.^[5]^

Current pathological classification recognizes AML as a member of the perivascular epithelioid cell tumor family – a distinct group of mesenchymal neoplasms exhibiting co-expression of melanocytic and smooth muscle markers.^[6]^ The 2022 World Health Organization classification system categorizes RAML as a renal mesenchymal tumor.^[7]^ While most RAMLs are benign, certain pathological subtypes demonstrate aggressive potential and vascular invasion.^[8]^ High-risk features included epithelioid variant showing malignant transformation potential, and documented cases of recurrence and distant metastasis.^[9]^

The decision to intervene in RAML depends on several clinical considerations including obvious symptoms or suspicion of malignant transformation.^[1]^ Special consideration should be given to women who are pregnant or planning pregnancy, as the estrogen-sensitive nature of these tumors may lead to accelerated growth during gestation.^[3,10]^ Various treatment modalities are available, ranging from minimally invasive approaches like arterial embolization and ablation to medical therapy with mammalian target of rapamycin (mTOR) inhibitors and definitive surgical resection.^[3]^

While embolization serves as the primary intervention for acute hemorrhage from spontaneous rupture-leveraging its ability to target the tumor’s characteristic hypervascularity – its utility is limited by the frequent occurrence of post-embolization syndrome (manifesting as fever and flank pain).^[1,3]^ This adverse effect profile generally precludes its routine use as a preoperative adjunct for giant tumors or hypovascular tumors. In the present case, the angiographic demonstration of multivessel tumor supply made embolization particularly effective. Beyond achieving immediate hemostasis, preoperative embolization offers several surgical advantages: it significantly reduces intraoperative blood loss, stabilizes the patient’s hemodynamic status, prevents renal venous congestion, and potentially decreases the risk of tumor cell dissemination during surgery.

Ablation therapy represents an effective option for small RAML, and mTOR inhibitors have become a mainstay for TSC-associated cases.^[3]^ The delayed therapeutic onset of mTOR inhibitors, combined with the uncertain efficacy in sporadic cases, rendered it unsuitable for our patient. The patient’s presentation with significant abdominal symptoms indicated imminent risk of spontaneous rupture and life-threatening hemorrhage, necessitating immediate intervention.

Currently, surgery remains the standard treatment for giant RAML. While nephron sparing surgery (NSS) has become the preferred approach for managing giant RAMLs to preserve renal function, RN remains necessary in specific clinical scenarios.^[1,3]^ RN is necessary under the following conditions: complete renal replacement by tumor; large or renal hilum tumor; suspected malignancy; when embolization is contraindicated or fails to control life-threatening hemorrhage.^[1,3]^ In our case, intraoperative findings revealed near-total renal parenchymal replacement by the tumor, rendering NSS technically unfeasible – minimal residual functional tissue after extensive resection would provide inadequate renal preservation. The substantial tumor dimensions and pronounced vascularity further contraindicated NSS due to the anticipated large surgical defect and significant hemorrhage risk. NSS retains its role as the procedure of choice when favorable anatomical conditions exist, including preserved renal architecture, extrahilar tumor location, and low suspicion of malignancy. For such vascular giant RAMLs, our experience confirms that embolization followed by timely surgical intervention optimizes outcomes while minimizing post-embolization syndrome risks.

A review of published cases involving RAML exceeding 30 cm in diameter reveals that 7 out of 8 reported patients underwent RN without preoperative embolization, while one case received no treatment (Table 1). Notably, these surgical interventions were consistently associated with substantial intraoperative blood loss, often necessitating transfusion support. In contrast, our patient who underwent preoperative arterial embolization experienced significantly reduced intraoperative bleeding and showed no postprocedural complications. While these findings suggest potential benefits of preoperative embolization for giant angiomyolipomas, the limited sample size and relatively short follow-up period in our study underscore the need for extended clinical observation and accumulation of additional cases to draw more definitive conclusions. Given the potential for RAML recurrence, a postoperative follow-up period exceeding 2 to 3 years is recommended for patients.^[1]^ A 1-year follow-up period is insufficient for RAML, as patients who underwent surgery have experienced metastasis or recurrence after a median follow-up of approximately 4 years.^[9]^ A recurrence rate of 3.4% was observed during the 8-year follow-up period among 58 patients,^[3]^ indicating the necessity of long-term monitoring.

**Table 1:** Giant renal angiomyolipoma in the indexed literature, by size.

Study	Year	Sex	Age (yr)	Size (cm)	Side	Etiology	Treatment	Blood loss
Katz DS	1997	Female	37	45 × 20 × 12 (L), 14.6 × 10 × 7.5 (R)	B	TSC	RN (L)	Uknown
Taneja R	2013	Female	49	39 × 25 × 9	L	NO	RN (L)	Unknown
Current case	2024	Female	35	36 × 26 × 13	L	NO	Embolism + RN (L)	Less
Bains L	2021	Male	26	35 × 20 × 12 (R), 14 × 6 (L)	B	TSC	NO	Unknown
Al-Hajjaj M	2021	Male	36	35	R	NO	RN (R)	1500 mL, input RBC 2 U
Nepple KG	2010	Male	53	35	R	NO	RN (R)	700 mL
Hussain M	2013	Female	34	32 × 16 × 12 (R), 27 × 21 × 16 (L)	B	TSC	RN (R)	Unknown
Ghaed MA	2020	Male	48	32 × 22 × 8 (L), 2 × 1 × 0.5 (R)	B	NO	RN (L)	Input RBC 1 U
Rosselló BM	2004	Male	40	31 × 19 × 10 (L), 3.5 (R)	B	TSC	RN (L)	A lot

B = both, L = left, R = right, RBC = red blood cell, RN = radical nephrectomy, TSC = tuberous sclerosis complex.

## 4. Conclusion

Giant RAMLs warrant active intervention due to the potentially life-threatening hemorrhage that may result from spontaneous rupture. While CT seves as the primary diagnostic modality, definitive diagnosis relies on immumohistochemical confirmation. For cases where the tumor has completely replaced the renal parenchyma, RN remains the treatment of choice. In the management of highly vascularized large tumors, preoperative embolization followed by prompt surgical intervention is recommended to minimize both intraoperative blood loss and the risk of post-embolization syndrome. The present case contributes valuable insights into the clinical management of giant sporadic RAMLs, offering a practical reference for clinicians encountering similar challenging cases. However, further accumulation of clinical experience through additional case studies will be essential to refine and optimize treatment strategies for these rare but clinically significant tumors.

## Author contributions

**Conceptualization:** Yu Wang.

**Funding acquisition:** Ting Yuan.

**Investigation:** Lingtao Yan.

**Methodology:** Xingzhi Li.

**Writing – original draft:** Lingtao Yan.

**Writing – review & editing:** Yu Wang.
